# A Pilot Study to Investigate the Feasibility of a Multiple Locus Variable Number Tandem Repeat Analysis to Understand the Epidemiology of *Dichelobacter nodosus* in Ovine Footrot

**DOI:** 10.3389/fvets.2020.581342

**Published:** 2020-12-02

**Authors:** Katharina Giebel, Laura E. Green, Kevin J. Purdy

**Affiliations:** ^1^School of Life Sciences, University of Warwick, Coventry, United Kingdom; ^2^School of Agriculture, Food and the Environment, Royal Agricultural University, Gloucestershire, United Kingdom; ^3^College of Life and Environmental Sciences, Institute for Microbiology and Infection, University of Birmingham, Birmingham, United Kingdom

**Keywords:** *Dichelobacter nodosus*, footrot, sheep, MLVA, veterinary epidemiology, bacteriology, PCR

## Abstract

*Dichelobacter nodosus* is the essential pathogen in ovine footrot, an important cause of lameness in sheep that reduces productivity and welfare. The aim of this study was to investigate the feasibility of using multiple locus variable number tandem repeat analysis (MLVA) developed to investigate isolates to understand the molecular epidemiology of *Dichelobacter nodosus* in ovine footrot by investigation of communities of strains. MLVA sensitivity was improved by optimizing PCR conditions to 100% specificity for *D. nodosus*. The improved MLVA scheme was used to investigate non-cultured DNA purified from swabs (swab DNA) and cultured DNA from isolates (isolate DNA) from 152 foot and 38 gingival swab samples from 10 sheep sampled on four occasions in a longitudinal study. Isolate DNA was obtained from 6/152 (3.9%) feet and 5/6 yielded complete MLVA profiles, three strains were detected. Two of the three isolate strains were also detected in isolate DNA from 2 gingival crevice cultures. Complete MLVA profiles were obtained from swab DNA from 39 (25.7%) feet. There were 22 *D. nodosus* community types that were comprised of 7 single strain and 15 multi-strain communities. Six community types were detected more than once and three of these were detected on the same four sheep and the same two feet over time. There were a minimum of 17 and a maximum of 25 strain types of *D. nodosus* in the study. The three isolate strain types were also the most frequently detected strain types in swab DNA. We conclude that the MLVA from swab DNA detects the same strains as culture, is much more sensitive and can be used to describe and differentiate communities and strains on sheep, feet and over time. It is therefore a sensitive molecular tool to study *D. nodosus* strains directly from DNA without culture.

## Introduction

Footrot (FR) is the most common cause of lameness in sheep in the UK and it is a health and welfare concern in sheep flocks globally (1–4). Footrot reduces productivity and sustainability of sheep farming (5, 6), costing the UK industry £20–£80 million per annum (6, 7).

There are two clinical presentations of footrot, interdigital dermatitis (ID), characterized by inflammation of the interdigital skin, and severe footrot (SFR), characterized by separation of the hoof horn from underlying tissues (8). The essential pathogen in footrot is the fastidious gram negative, aerotolerant, anaerobic bacterium *Dichelobacter nodosus* (9, 10) which is key in initiation of ID and in progression to SFR (11–13). *D. nodosus* is present in >90% of UK sheep flocks and causes ~70 % of lameness (4).

In cross-sectional studies, *D. nodosus* has been detected on healthy and diseased feet (11, 14–18), and in the gingival cavity (19).

Russell et al. (20) developed an MLVA assay as a strain-typing tool for cultured *D. nodosus* isolates based on four polymorphic loci (DNTR02, 09, 10, and 19). The assay was used by Smith et al. (21) to investigate within-flock population dynamics of strains of *D. nodosus*. They reported that *D. nodosus* strains clustered within sheep and were transmitted between ewes over time. *D. nodosus* isolation is challenging and time consuming, because of the organism's fastidious and anaerobic nature and direct PCR from swab DNA is more sensitive than culture (15, 22).

Muzafar et al. (23) used the MLVA developed by Russell et al. (20) to analyse *D. nodosus* directly from DNA extracted from interdigital skin swabs of healthy and footrot affected feet using only DNTR10 and DNTR19. They did not use DNTR09 due to poor amplification nor DNTR02 due to non-specific amplification.

Therefore, the aim of this study was to optimize and validate the full *Dichelobacter nodosus* MLVA for isolate DNA and community DNA and investigate its value in a pilot longitudinal study of persistence of *D. nodosus* in sheep.

## Materials and Methods

### Sample Collection

In 2014, swab samples were collected in a longitudinal study of 10 sheep (5 ewes, 5 lambs) from a UK flock with footrot. All sheep were sampled on four occasions at 2-week intervals from May to June. On each occasion, all four feet and the gingival crevice were swabbed with two swabs each, one for DNA analysis and one for culture. In addition, foot lesions were scored for ID and SFR using two 5-point scales (15). A total of 152 foot and 38 mouth swabs were collected.

### Isolation and Detection of *Dichelobacter nodosus*

Swabs for culture were inoculated onto 4% hoof agar (HA) followed by subculture onto a 2% HA (15). Plates were incubated under anaerobic conditions at 30°C for 4–5 days (MACS-MG-1000 anaerobic workstation, Don Whitley Scientific, Shipley, UK, 80% N_2_, 10% CO_2_, 10% H_2_). Isolate DNA was extracted with a DNeasy Blood and Tissue Kit (Qiagen Ltd., Manchester, United Kingdom) according to the manufacturer's instructions with a lysis time of 1 h. DNA was extracted directly from swabs using the hydroxyapatite spin-column method (24) using only 0.5 ml of the sodium phosphate extraction buffers. *D. nodosus* was detected in DNA extracted from the foot and mouth swabs using a *D. nodosus*-specific *rpoD*-targeted qPCR (25).

### MLVA Optimization and Protocol

The sensitivity of the MLVA primers (Supplementary Table 1) was determined using DNA from *D. nodosus* isolates and swab samples. Improvements in sensitivity were made by changing the PCR Master mix (From Promega x2 PCR Master Mix to Bioline MyTaq™ Red Mix), increasing primer concentration (from 10 pmoles of each primer in a 50 μl reaction to 10 pmoles of each primer in a 25 μl reaction), DNA template concentration (from 1 μl/50 μl reactions to 1 μl/25 μl reactions) and the number of PCR cycles (from 30 to 40 cycles). The final protocol was, in 25 μl reactions; 12.5 μl MyTaq™ Red Mix (Bioline, London, United Kingdom), 1 μl of each primer (10 μM stock concentration; Supplementary Table 1), 1 μl bovine serum albumin (20 mg ml^−1^) (Sigma Aldrich, Dorset, United Kingdom) and 1 μl of DNA template. DNA from *D. nodosus* strain 4303 LBV and nuclease free H_2_O were used as positive and negative controls, respectively. All PCR reactions were carried out on an Eppendorf Mastercycler ep gradient machine (Eppendorf, Hamburg, Germany) using the following cycling conditions: One cycle of 95°C for 2 min, 40 cycles of 95°C for 1 min, 59°C for 30 s and 72°C for 1 min with a final extension of 72°C for 2 min. PCR products were visualized by ethidium bromide-stained agarose gel electrophoresis and imaged using a Gene Flash imager (Syngene Bio Imaging, Cambridge, United Kingdom).

### Determination of VNTR Amplicon Size Using Fragment Analysis

MLVA amplicon size was determined using fragment analysis. Forward primers for the four loci were labeled with different fluorescent dyes (Supplementary Table 1) and amplicons for each locus were submitted separately for fragment analysis (DNA Sequencing and Services, University of Dundee, Scotland). PCR products from *D. nodosus* isolates were diluted 1:100 and products originating from swabs were diluted either 1:20 or 1:100 depending in the PCR band intensity seen on agarose gel. 1200 Liz dye (Applied Biosystems, Warrington, United Kingdom) was used as a size standard and data were analyzed using Peak Scanner? Software (Applied Biosystems, Warrington, United Kingdom). The bin range was set to 4bp (fragment size ± 2 bp) and minimum fragment length cut off values 500, 500, 400, and 550 bp for DNTR02, 09, 10, and 19, respectively, based on the length of each fragment without repeats.

To provide additional information on the accuracy of the assay, the 4 loci were amplified from *D. nodosus* strain 1703A (GenBank Accession number CP000513) and submitted for fragment analysis and Sanger sequencing. The size of the loci from fragment analysis was compared with the published 1703A sequences for the 4 loci (Genebank Accession numbers KC676717, KC676718, KC676719, and KC676720 for DNTR02, 09, 10, and 19, respectively). The sequenced VNTR loci from *D. nodosus* strain 1703A were analyzed using tandem repeat (TR) finding software (26) to determine the numbers of repeats in the sequence.

MLVA analysis of two isolates resulted in a primary peak and a number of small secondary peaks ≤ 20% the height of the primary peak at expected TR intervals (Supplementary Figure 1). To investigate secondary peaks, 14 more *D. nodosus* isolates were analyzed (Supplementary Table 2). Secondary peaks were observed in some, but not all, isolates. The secondary peaks might have been due to non-axenic *D. nodosus* cultures. However, this is unlikely because multiple peaks were detected at all 4 loci (Supplementary Figure 1) which would indicate many isolate strains in each culture. Other explanations for secondary peaks might be rapid, minor, within-strain variation or an artifact in the PCR process. It was not possible to adjust the PCR to prevent the formation of these small secondary peaks, therefore it was decided to consider secondary peaks ≤ 20% the height of the primary peak as artifacts (Supplementary Figure 2).

### Validation of the MLVA PCR and Testing on Model Communities

Primer specificity was tested by MLVA analysis of DNA from non-target organisms previously detected on sheep feet or present in soil or feces. These were *Streptococcus uberis, Staphylococcus epidermis, Staphylococcus intermedius, Staphylococcus aureus, Staphylococcus hyicus, Staphylococcus chromogenis, Streptococcus dysgalactidae, Streptococcus agalactidae, Mannheimia spp., Fusobacterium necrophorum, Pseudomonas aeruginosa, Escherichia coli*, and *Mycobacterium tuberculosis*.

To confirm specificity PCR products from 10 *D. nodosus* positive samples from foot swabs and 1 gingival swab were analyzed using MLVA and the resulting VNTR amplicons were submitted for Sanger sequencing (GATC Biotech AG, Cologne, Germany). Sequences were assessed for quality using CodonCode Aligner version 6.0.2. and analyzed using BLAST (27).

MLVA sensitivity was investigated by assessing its limit of detection of *D. nodosus* load. *D. nodosus* strain 4303 LBV was incubated on Eugon Agar (28) for 5 days. After incubation 1 ml of PBS, pH 7.4, was added to the *D. nodosus* plates, creating a cell suspension. Suspended cells were quantified using a Petroff-Hausser counting chamber (Hausser Scientific, PA, USA) and serially diluted. Sterile swabs were spiked with 50 μl of the serially diluted cell suspension resulting in 1.07 × 10^6^ to 1.07 *D. nodosus* cells per swab (equivalent to *rpoD* copies per sample). DNA was extracted from these swabs as above. Samples were then screened for *D. nodosus* using the MLVA assay. To test whether detection could be improved further, all samples were submitted to a second round of the MLVA PCR assay using the same cycling conditions.

The feasibility of using MLVA to analyse multistrain communities was investigated using model communities created from eight *D. nodosus* isolates with different MLVA profiles. Two model communities (1 and 2) were created, each with 4 isolates (Table 1). DNA from each isolate was standardized to a concentration of 15 ng/μl and 5 μl of DNA from each isolate mixed in a 1:1:1:1 ratio. A third model community was created with the isolates in community 1, with one isolate (*D. nodosus* 1703A) diluted 5-fold to investigate whether non-dominant isolates could be detected. These model communities were amplified and analyzed using the MLVA protocol above.

**Table 1:** Input and recovery of MLVA PCR amplicons and peak sizes in *Dichelobacter nodosus* isolates and model communities.

***D.nodosus* isolates**	**Size (bp)**	Peak size (RFU)^*^		**Size (bp)**	**Peak size (RFU)**		**Size (bp)**	**Peak size (RFU)**		**Size (bp)**	**Peak size (RFU)**	
		**DNTR02**			**DNTR09**			**DNTR10**			**DNTR19**	
		**Individ**	**Comm**		**Individ**	**Comm**		**Individ**	**Comm**		**Individ**	**Comm**
**Community 1**												
VCS 1703A	545	15,530	209^**^	985	11,722	2,323^**^	693	9,795	182^**^	851	7,855	219^**^
JIR3918	610	8,091	2,950	768	28,924	16,045	835	22,117	483^**^	1,019	3,692	1,621
JIR3919	650	13,779	2,681	876	18,892	11,636	646	10,793	101^**^	1,019	2,078	1,621
JIR3350	560	16,543	4,975	985	23,681	12,323	505	25,910	8,144	932	6,326	1,178
**Community 1 (diluted)**												
VCS 1703A (1:5)	545	13,092	85^**^	985	11,812	1,205^**^	693	5,382	138^**^	851	8,624	372^**^
JIR3918	610	8,091	4,034	768	28,924	16,045	835	22,117	1,158	1,019	3,692	3,384
JIR3919	650	37,779	4,501	876	18,892	11,363	646	10,793	3,786	1,019	2,078	3,384
JIR3350	560	16,543	1,597	985	23,681	1,205^**^	505	25,910	3,030	932	6,326	242^**^
**Community 2**												
VCS 1703A	545	15,530	150^**^	985	11,812	5,194	693	5,382	339^**^	851	8,627	129^**^
JIR3918	610	8,091	1,874	786	28,924	10,421	835	22,117	2,322	1,019	3,692	872
4303 LBV	635	9,853	705^**^	985	19,638	5,194	788	/^***^	687	1,019	2,714	872
BS8	555	18,255	4,529	985	9,345	5,194	835	3,475	2,322	933	5,191	1,814

* *Peak size (RFU), the size of the peak observed in relative fluorescent units; Individ, peak size for a single strain; Comm, Peak size of the strain in a mixed model community*.

** *Recovered products that fall below the established threshold*,

*** *No PCR product detected*.

### Assessment of the MLVA Assay on DNA Extracted From Foot and Gingival Swab Samples

**Figure 1:**
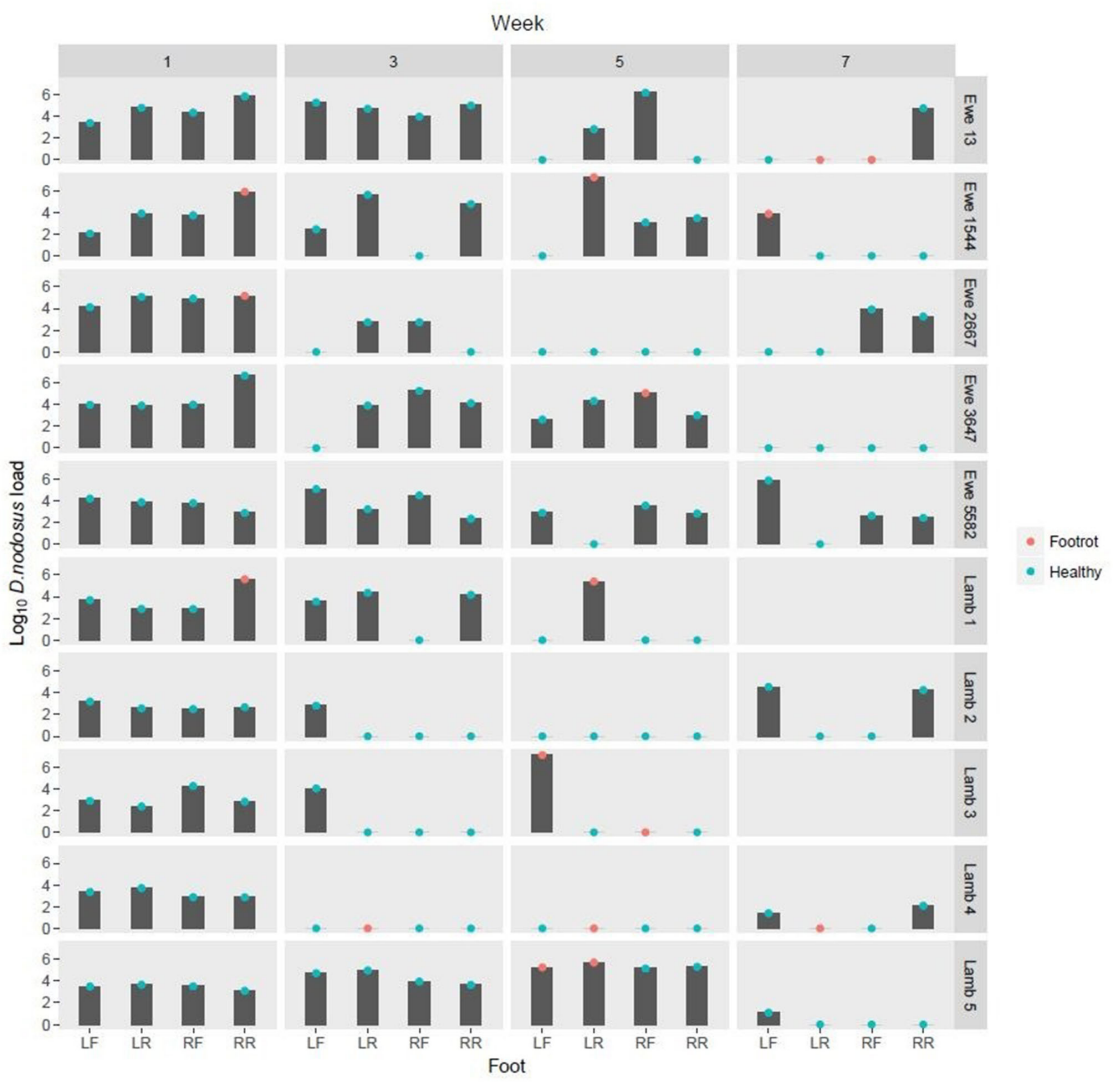
Log_10_ *Dichelobacter nodosus* load on DNA swabs from feet from weeks 1, 3, 5, and 7. Green dots: Healthy foot (Interdigital dermatitis score 0 or 1, footrot score 0) Red dots: Foot classed as diseased with footrot (Interdigital dermatitis score >1 and/or footrot score >0) LF, Left front; LR, left rear; RF, Right front; RR, Right Rear.

Swab DNA and isolate DNA from feet and gingivae from the longitudinal study that were positive for *D. nodosus* by *rpoD* qPCR were analyzed using MLVA and fragment analysis. Secondary peaks ≤ 20% the height of the primary peak were excluded (Supplementary Figure 2). Distinct community fingerprints were obtained that could be compared visually (Figure 1). In addition, strain types within a community could be defined as “definitely present” when a community had only one variant at each locus or when more than 1 variant was detected at one locus only and one variant at the other three loci. When there were 2 or more variants at 2 or more loci strains were defined as “possibly present.” The minimum and maximum number of strain types in a community were calculated using the formulae:

$$\begin{array}{l} Minimum\;number\;of\;strains\\ \;=number\;of\;variants\;at\;the\;most\;variable\;locus\\ Maximum\;number\;of\;strains\\ \;=product\;of\;the\;number\;of\;variants\;at\;each\;locus \end{array}$$

## Results

### Validation and Optimisation of the MLVA Scheme

The number of TRs at the four loci for strain 1703A was the same as reported by Russell et al. (20). The MLVA assay was specific for *D. nodosus* with no amplification in any of the 4 loci in non-target species (Supplementary Figure 3). DNA sequencing of the amplified products from foot swabs were 99–100% similar to their target sequences confirming specificity of the MLVA PCR. The mouth swab DNA yielded products for three MLVA loci, DNTR02, 09 and 10, which were 96–97% similar to their target sequence. The improved detection limit of the MLVA protocol after a single round of PCR was 10^2^,10^3^, 10^2^, and 10^3^ copies μl^−1^ DNA template for DNTR02, DNTR09, DNTR10, and DNTR19, respectively. A second round of MLVA PCR resulted in non-specific amplification and diluting samples did not improve specificity or sensitivity (data not shown).

#### VNTR Amplicons From *Dichelobacter nodosus* Model Communities

All *D. nodosus* strains in the model communities were detected, including community 3 where one strain was at 5 fold dilution; 17/48 secondary peaks were ≤ 20% the height of the primary peak and excluded (Table 1); 11 of these 17 were from the VCS strain, which was only detected correctly once, suggesting that this laboratory strain is particularly difficult to detect using MLVA.

#### Longitudinal Study of Persistence of *D. nodosus* in the Epidemiology of Footrot

FR was detected at least once on the feet of 8/10 sheep (Figure 1). *D. nodosus* was detected by qPCR in 97/152 (63.8%) foot swab DNA samples. It was detected on both healthy and diseased feet and on all sheep, but not all weeks (Figure 1).

**Figure 2:**
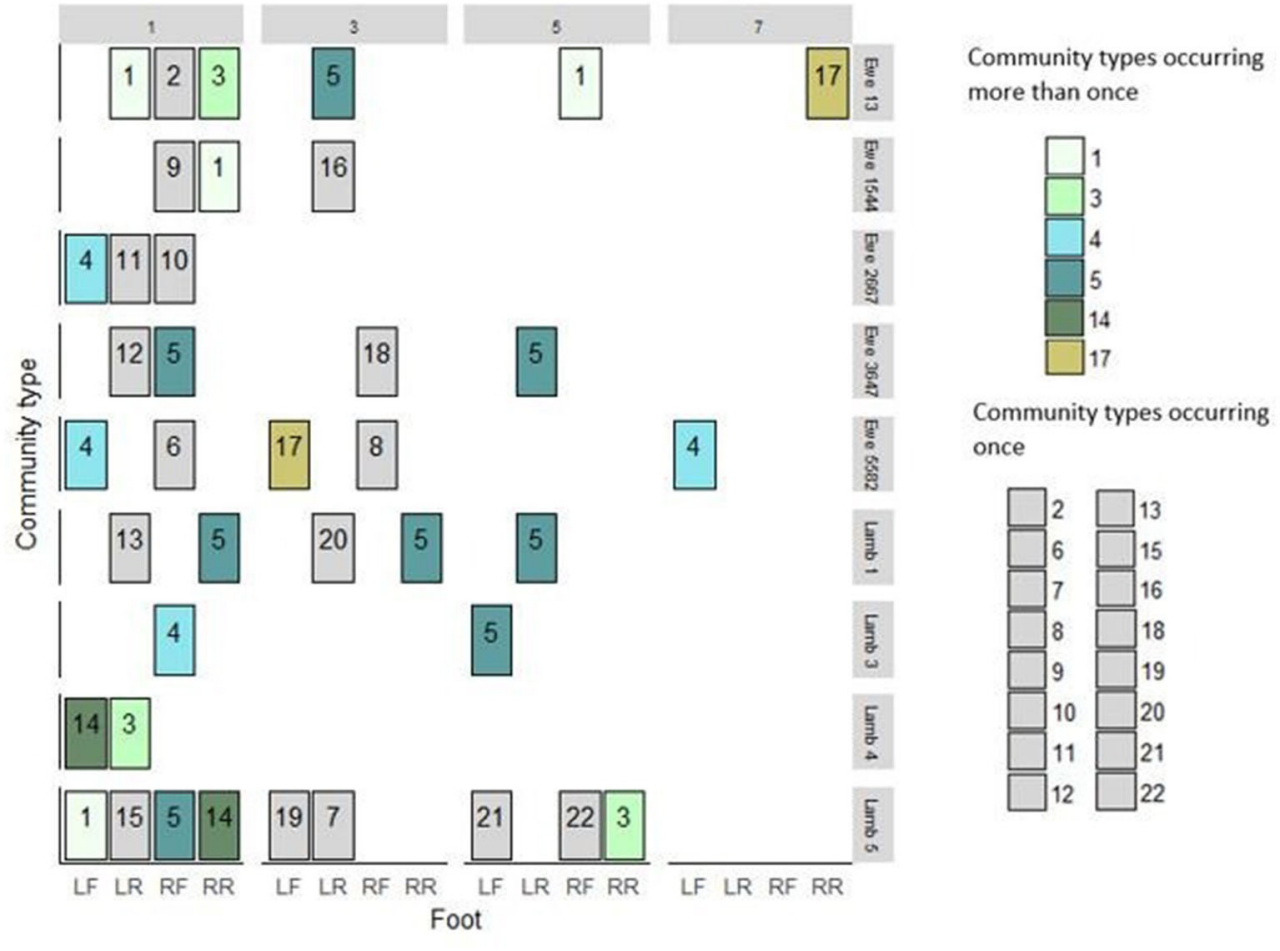
*Dichelobacter nodosus* community profiles from 39 feet of 9 sheep from weeks 1, 3, 5, and 7. Community types in green shades were detected repeatedly. Community types in gray occurred only once in the study. LF, Left front; LR, left rear; RF, Right front; RR, Right Rear.

**Figure 3:**
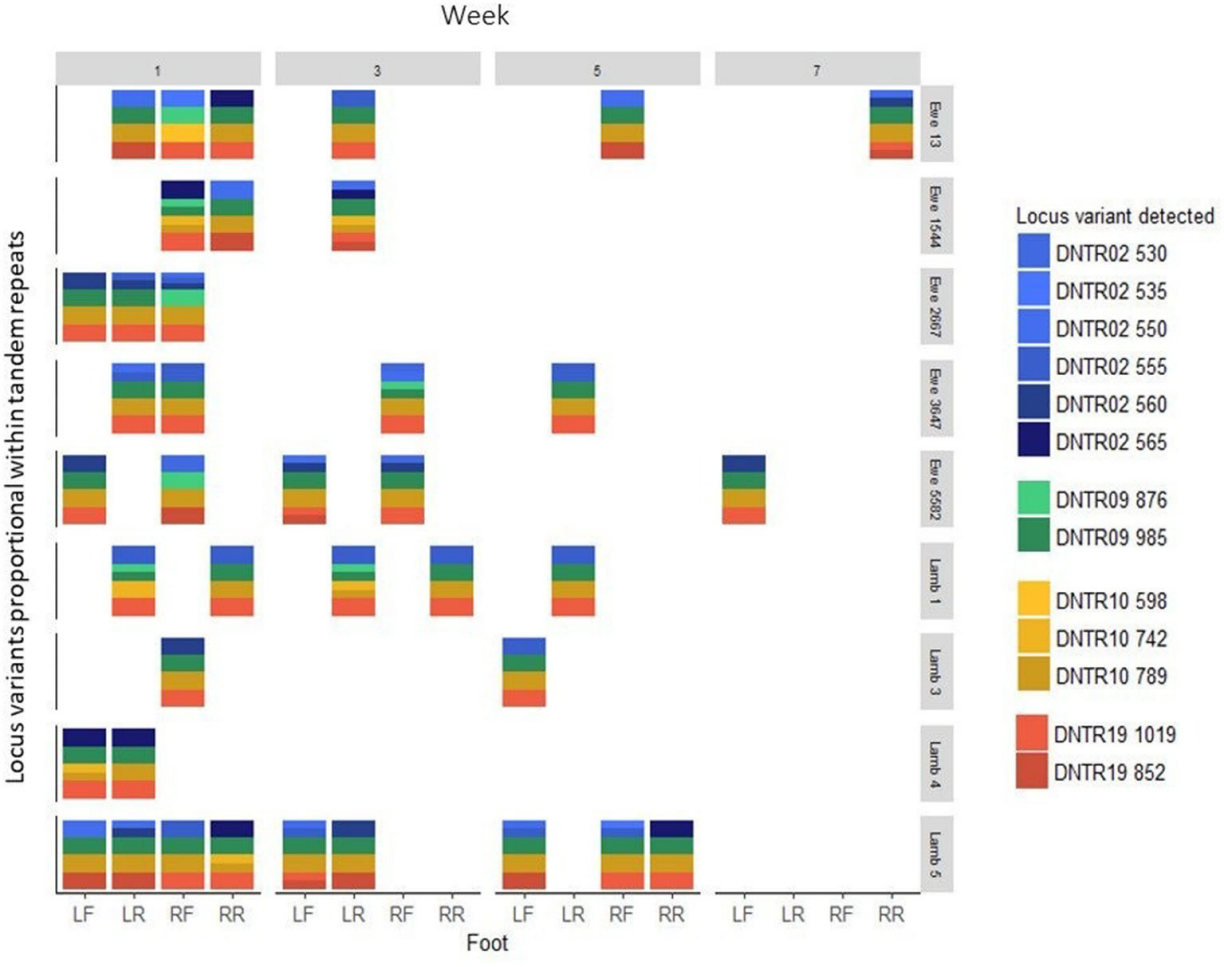
*D. nodosus* MLVA DNTR = *D. nodosus* tandem repeat 02, 09, 10, 19, variants proportional within TR from DNA from swab samples from 39 feet from 9 sheep in weeks 1, 3, 5, and 7. LF, Left front; LR, left rear; RF, Right front; RR, Right rear. Four colors, each of one shade, indicates a single strain.

Out of the 97 *D. nodosus* positive foot swab DNA samples, 53 (54.6%) amplified all 4 MLVA loci and in 39/53 (73.6%) samples complete MLVA profiles were obtained (Figure 2). The 39 complete profiles had a total of 156 loci with 75/156 (48.1%) with secondary peaks. The total number of peaks in these 156 loci was 220. After application of the ≤ 20% threshold (Supplementary Figure 2) 106/220 (48.2%) peaks were excluded from further analysis. DNTR02 was the most variable locus with 6 TRs and DNTRs 09 and 10 were the least variable with only 2 TRs (Figure 3).

*D. nodosus* was detected by qPCR in 8/38 (21.1%) gingival swab DNA samples, however, no complete MLVA profiles were obtained. Three loci were amplified in one sample and these matched loci in community types 3 and 4.

#### *Dichelobacter nodosus* Community Profiles From Foot Swab DNA

There were more MLVA positive feet in week 1, and detections declined over the 4 visits (Figure 3). There were 22 *D. nodosus* community types in total; seven were single strains and fifteen were multistrain; six community types were detected more than once, three of these were detected on the same four sheep and the same two feet over time (Figure 2). Community type 5, a single strain community, was most frequently detected (8/39 feet) (Figure 2). The results indicate that the MLVA differentiated *D. nodosus* communities spatially between feet, sheep, and over time.

#### *Dichelobacter nodosus* Strain Type Analysis From Foot Swab DNA

There were 17 strains definitely present and a further 8 possibly present in the 22 *D. nodosus* community types in foot DNA (Table 2). Seven strains were, definitely or possibly, present on more than 4 occasions and the remaining 18 strains were present on 1 to 3 occasions. The three most frequently detected single strains were C, D and E (Table 2), these were the single strain community types 3, 4, 5 (Figure 2).

**Table 2:** MLVA *Dichelobacter nodosus* strain types from swab DNA from the feet of sheep by definitely and possibly present.

**MLVA strain type**	**Frequency definitely present**	**Frequency possibly present**
A	4	2
B	1	2
C^*^	10	12
D^*^	7	9
E^*^	8	5
F	2	5
G	2	8
H	5	10
I	1	4
J	1	1
K	0	1
L	0	1
M	1	2
N	1	1
O	1	1
P	0	1
Q	1	2
R	1	1
S	0	1
T	0	1
U	0	1
V	0	2
W	1	2
X	0	1
Y	1	1

* *Strain also detected in culture isolates*.

#### *Dichelobacter nodosus* Strains and Communities From Isolate DNA

*D. nodosu*s was cultured from 6/152 (3.9%) foot swabs from 3 sheep in weeks 1, 3, and 5, with 5 complete and 1 partial MLVA profile obtained from these isolates. The three most frequently detected strains, C, D, and E (Table 2), were also detected in swab DNA from the same foot at the same time (Table 3). Complete MLVA profiles were obtained from 2 isolates from the gingival crevice and *D. nodosus* strains C and D (Community types 3 and 4) were detected in both samples (Table 3).

**Table 3:** MLVA profile of *Dichelobacter nodosus. nodosus* from isolate DNA compared with swab DNA.

**Sample origin**	**Sheep ID**	**Week of study**	**Locus fragment size/number of repeats**				**Isolate DNA strain type**	**Swab DNA strain types**
Foot			DNTR02	DNTR09	DNTR10	DNTR19		
RR	13	1	565/10	985/5	789/8	1,019/5	C	C
LR	3,647	1	555/8	985/5	789/8	1,019/5	C	E, C^**^
RF	3,647	1	555/8	985/5	789/8	1,019/5	E	E
LF	5,582	1	560/9	985/5	789/8	1,019/5	D	D
RR	13	3	565/10	985/5	789/8	1,019/5	C	NS
LR	13	3	555/8	985/5	789/8	/	E^*^	E
Mouth	13	3	560,565/9–10	985/5	789/8	1,019/5	C, D	NS
Mouth	3	3	560,565/9–10	985/5	789/8	1,019/5	C, D	∧

*RR, Right rear; RF, Right front; LR, Left rear; LF, Left front*.

* *DNTR19 did not amplify, but strain type E present in corresponding swab*.

** *E, C = both strain types are definitely present, NS, no swab data for all 4 loci, ∧DNTR19 did not amplify, other VNTR's identical*.

## Discussion

The optimized *D. nodosus* MLVA (20) was developed and used successfully to investigate *D. nodosus* isolates and communities from DNA extracted directly from swab samples. The variability in loci and community types in the longitudinal study indicate that the improved MLVA scheme is more sensitive than previous studies (21, 23) and can be used to improve understanding of the epidemiology of communities of *D. nodosus* on feet over time.

Smith et al. (21) investigated transmission and persistence of *D. nodosus* strains on sheep's feet over a 10 months period using the original assay (20) on isolates of *D. nodosus*. They reported 45 animal-level repeat *D. nodosus* isolation events, 47% of those were isolation events of the same strain from the same foot over time. In addition, they detected and isolated the population dominant strain. In this, albeit shorter study, we obtained similar results with 4 animal-level repeat detection of *D. nodosus* communities and one dominant single strain community (community 5) that was detected on the same foot consecutively.

There was a high level of variability in *D. nodosus* communities in our study, with most variation occurring in week 1. This is possibly attributable to rainfall on and preceding the day of sampling as wet weather facilitates persistence of *D. nodosus* (29). A high level of *D. nodosus* variability was also reported by Smith et al. (21) who isolated 87 MLVA types over 10 weeks, which suggest that a large number of strains are present in footrot affected flocks and suggest that our findings represent true variability.

Our optimized MLVA was used in a subsequent longitudinal study to investigate persistence of *D. nodosus* strains on feet; this demonstrated that *D. nodosus* strains persist on the feet of diseased sheep, but not on the feet of healthy sheep (29).

Only 6 isolates were cultured in this study using published *D. nodosus* isolation techniques, however, the strain types from culture isolates were a subset of the strains detected from the non-culture DNA (Table 3) at the same site and time, indicating that MLVA analysis of non-cultured DNA is more sensitive than DNA from culture, as reported by others for other bacterial species (30–32). This indicates that previous studies that have compared relationships between *D. nodosus* using MLVA profiles of isolates (21) are incomplete and analysis would have been improved using MLVA from DNA directly.

The MLVA scheme had a limit of detection of ~10^3^ genome copies μl^−1^ of extracted DNA and so there were a number of samples that were positive for *D. nodosus* by qPCR and negative by MLVA. Therefore, even the MLVA is not 100% sensitive. Despite this, the ability to use MLVA on non-cultured DNA to identify strains as definitely and possibly present and to produce fingerprint profiles is novel and adds to the value of MLVA as a tool to investigate strains of *D. nodosus* on feet over time.

This is the first occasion that a *D. nodosus* strain profile has been obtained from the gingival crevice, although complete strain profiles were detected from isolates and only incomplete strain profiles were obtained directly from swab DNA. Strain types C and D were present in mouths and were also among the strains most frequently detected on feet (Table 3).

The presence of regular secondary peaks in fragment analysis when testing isolate DNA has not been reported previously, although artifactual DNA extension during PCR has been reported but usually only for short sequences (33, 34). Further investigation of the secondary peaks was outside the scope of the current study and so a conservative threshold was applied to decrease the probability of artificially increasing the number of loci in a sample. As a consequence, it is possible that some non-dominant strains that were present at low abundance were classified as artifacts.

## Conclusions

A sensitive and specific *D. nodosus* MLVA assay using four VNTR loci was validated and optimized for use on non-culture DNA. The strain types detected from isolate DNA from the same site were a subsample of those detected from swab DNA, but many more strains were present in swab DNA, indicating that it is more sensitive to analyse *D. nodosus* from DNA directly rather than via culture. Because the MLVA can be used to identify communities of *D. nodosus* on the feet of sheep over time it can be used to investigate persistence and transmission of communities of *D. nodosus* and so improve epidemiological understanding of *D. nodosus*. Other MLVA schemes may be developed for use in the non-culture based study other bacterial species.

## Data Availability Statement

The raw data supporting the conclusions of this article will be made available by the authors, without undue reservation.

## Ethics Statement

The animal study was reviewed and approved by Animal Welfare & Ethical Review Body (AWERB), University of Warwick.

## Author Contributions

The work presented in this article was conducted by the KG. LG and KP supervised the research. All authors contributed to the article and approved the submitted version.

## Conflict of Interest

The authors declare that the research was conducted in the absence of any commercial or financial relationships that could be construed as a potential conflict of interest.
