# Supplementary material for: A Pilot Study to Investigate the Feasibility of a Multiple Locus Variable Number Tandem Repeat Analysis to Understand the Epidemiology of Dichelobacter nodosus in Ovine Footrot
Source: Front Vet Sci. 2020 Dec 2;7:581342. doi: 10.3389/fvets.2020.581342 (PMC7738329; doi:10.3389/fvets.2020.581342)
Supplement: Supplementary file 1 [file Data_Sheet_1.docx]

Supplementary Material

# Supplementary Tables

**Supplementary table S1** Variable number tandem repeat loci for *Dichelobacter nodosus* reported by Russell *et al*. (2014), primers and associated fluorescent dyes

| VNTR/  primer name | Primer sequence (5’– 3’) | Dye name | Dye colour |
| --- | --- | --- | --- |
| DNTR02 | Forward: GAT CCA TCG TTT CAT CGT CA (FL)*  Reverse: CGC ACT TTA GCC GTT ATG TTT | 6-Fam | Blue |
| DNTR09 | Forward: GGC GTA AAC GAA ATG CCT AA (FL)  Reverse: ATC GGC GGA AGA TTG TCT C | Vic | Green |
| DNTR10 | Forward: CCG TCT ATC CAC CCG ATT TA (FL)  Reverse: TTG AAC CGC GTC ACT ATC AG | Net | Yellow |
| DNTR19 | Forward: CCC GTC GAA TCA CTC CAG (FL)  Reverse: GGT AGC GCC GAA GAA AGA | Pet | Red |

* FL = 5’fluorescent labelled

**Supplementary table S2** *Dichelobacter nodosus* strains used to investigate the presence of minor peaks seen in traces after fragment analysis and to create model communities.

| Organism/Strain id. | Country of isolation | Source | Virulence | Serogroup |
| --- | --- | --- | --- | --- |
| VCS 1703A | Australia | J.R. Egerton, University of Sydney | Virulent | G |
| 4303 LBV | UK | University of Warwick | Virulent | unknown |
| C305 | Australia | J.R. Egerton, University of Sydney | Benign | H |
| UNE135 | Australia | B.F. Cheetham, University of new England | Benign | unknown |
| UNE149 | Australia | B.F. Cheetham University of new England | Virulent | unknown |
| CS101 | Australia | D.J. Steward, CSIRO*, Parkville | Benign | unknown |
| VCS1690 | Australia | J.R. Egerton, University of Sydney | Unknown | H |
| 13295C | Australia | Wagga Wagga University | Unknown | F |
| VCS1001 (A198) | Australia | J.R. Egerton, University of Sydney | Virulent | A |
| SP-02-418-C | Spain | Unknown | Virulent | E |
| SP-02-428-C | Spain | Unknown | Virulent | E |
| SP-02-473-C | Spain | Unknown | Virulent | E |
| SP-02-475-C | Spain | Unknown | Virulent | E |
| SP-02-508-C | Spain | Unknown | Virulent | E |
| SP-02-520-C | Spain | Unknown | Virulent | E |
| BS8 | UK | L.J. Moore, University of Bristol | Virulent | H |

All organisms are ovine isolates taken from the feet of sheep.

## Supplementary Figures


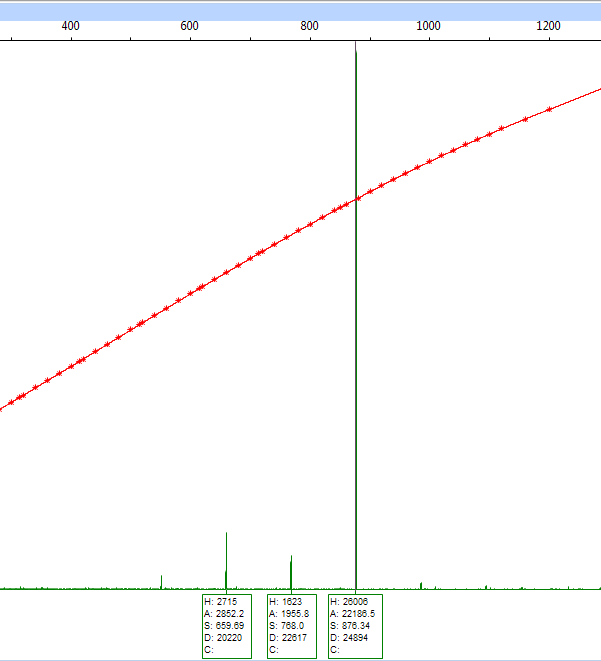


🡸Expected peak:

877 bp

*n*=4

Unexpected peaks


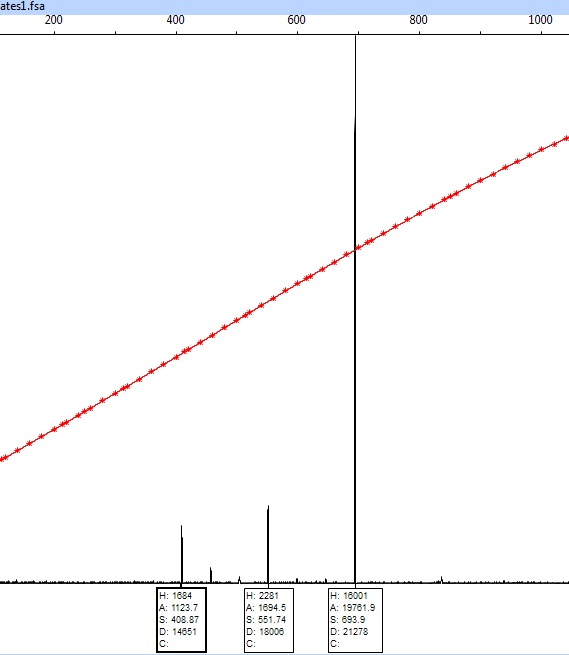


🡸Expected peak:

693 bp

*n*=6*

Unexpected peaks

n= 0 1 2 3 4 5

A: DNTR10

B: DNTR09

n= 1 2 3


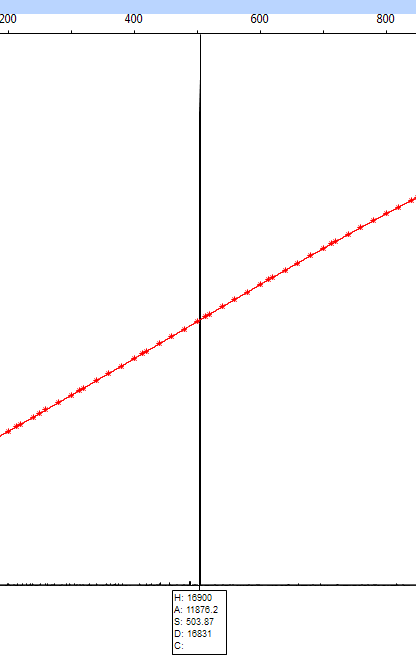


C: DNTR10

**Supplementary Fig. S1**: Electropherogram of the DNTR10 locus of *Dichelobacter nodosus* isolate VCS1703A (**A**) and the DNTR09 locus of isolate UNE149 (**B**) after MLVA and fragment analysis. A range of unexpected peaks were observed. All peaks are multiples of 48 bp (DNTR10) and 108bp (DNTR09), corresponding to the sizes of the tandem repeats. **C**: Electropherogram of the DNTR10 locus of the Australian *D. nodosus* isolate VCS1690. No unexpected peaks were observed. * **n**= number of tandem repeats that correspond to the peak

**
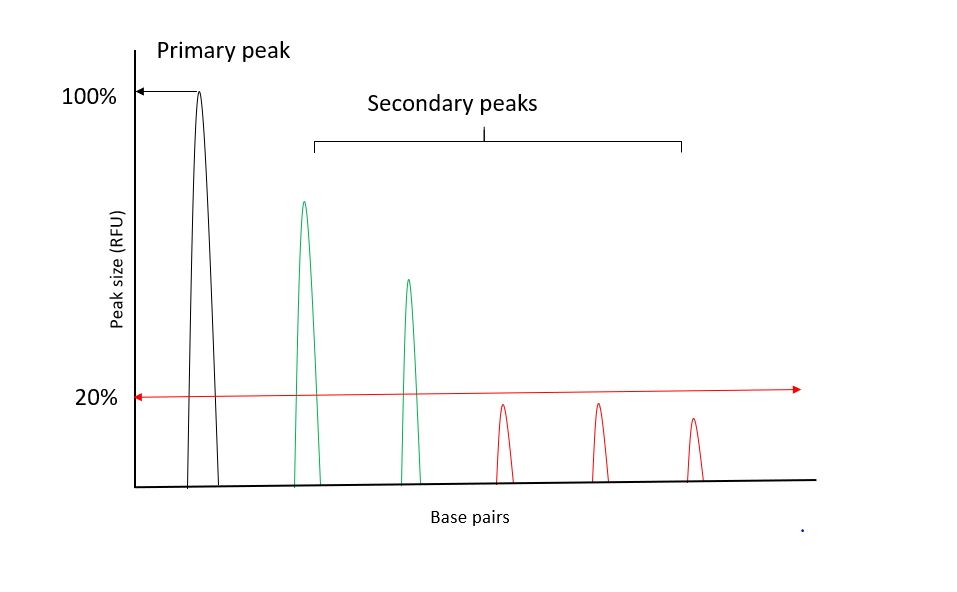
Supplementary Fig. S2** Diagram showing how the >=20% threshold was applied to peaks in an electropherogram after fragment analysis. The application of the threshold is relative to the size of the primary peak. Green secondary peaks are above the threshold and would be included in the analysis. Red peaks fall below the threshold and would be excluded. In this study there were only 7/75 [9.3%] electropherograms with secondary peaks that were ± 5% of the ≤ 20% threshold, resulting in a clear divide between secondary peak heights above and below the threshold). Secondary peaks above the threshold ranged from 21.6%-94.4% (median=52.8%) of the primary peak while secondary peaks below the threshold ranged from 1.5%-17.1% (median=6.2%) of the primary peak.


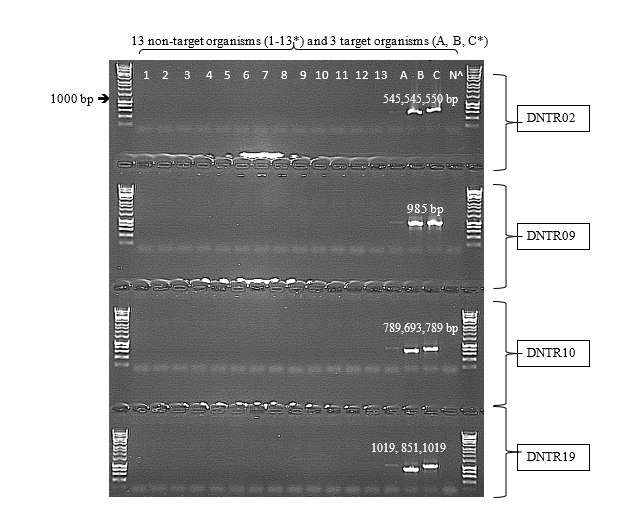


**Supplementary Fig. S3** Test of non-target organism for the *Dichelobacter nodosus* MLVA assay shown on 1% (w/v) agarose gel. **^N**= Sterile water non-template control. Bioline 1 kb DNA ladder with 1000 bp marker indicated. Note that amplification of control strain C305 (lane A) is weak probably due to low DNA concentration in the sample. Legend of organisms: **1** *Staphylococcus uberis,* **2** *Staphylococcus epidermis,* **3** *Staphylococcus intermedius,* **4** *Staphylococcus aureus,* **5** *Staphylococcus hyicus,* **6** *Staphylococcus chromogenis,* **7** *Streptococcus dysgalactidae*, **8** *Streptococcus agalactidae,* ***9*** *Mannheimia spp.,* **10** *Fusobacterium necrophorum,* **11** *Pseudomonas aeruginosa,* **12** *Escherichia coli,* **13** *Mycobacterium tuberculosis,* **A** *D. nodosus* strain C305*,* **B** *D. nodosus strain* VCS1703A*,* **C** *D. nodosus* 4303 LBV.
